# Investigation of the Effects of Acacetin on Autophagy Pathway and Exosome Release in Amyloid Beta Peptide-Induced Toxicity Models

**DOI:** 10.1007/s12035-025-04908-3

**Published:** 2025-04-21

**Authors:** Nilufer Ercin, Nail Besli, Bahar Sarikamis Johnson, Rabia Kalkan Cakmak, Merve Beker, Mustafa C. Beker, Ulkan Celik

**Affiliations:** 1 Department of Medical Biology, Hamidiye School of Medicine, University of Health Sciences, Istanbul, Turkey; 2 Department of Medical Biology, Hamidiye International School of Medicine, University of Health Sciences, Istanbul, Turkey; 3 Department of Physiology, School of Medicine, Istanbul Medeniyet University, Istanbul, Turkey; 4 Research Institute for Health Sciences and Technologies (SABITA), Regenerative and Restorative Medicine Research Center (REMER), Istanbul Medipol University, Istanbul, Turkey; 5 Department of Medical Biology, Institute of Health Sciences, University of Health Sciences, Istanbul, Turkey

**Keywords:** Acacetin, Alzheimer’s disease, Autophagy, Exosomes, Molecular modeling

## Abstract

Understanding   the mechanism behind Alzheimer’s disease is imperative due to the critical role of the autophagy pathway in protein homeostasis and neuronal survival. Autophagy pathway irregularities in neurons may increase exosome-mediated toxic protein transport, which can spread neurodegenerative diseases. Compelling evidence hints that acacetin (ACA) is a naturally occurring biocomponent exhibiting neuroprotective pharmacological properties. However, further molecular investigations are pressing to uncover the therapeutic potential of ACA. The present investigation endeavors to scrutinize the impact of ACA on the autophagy pathway and exosome release in an amyloid beta (Aβ) peptide-induced toxicity model. Herein, first, molecular modeling was performed between ACA and autophagy-related proteins. Afterward, the Aβ peptide-induced toxicity model cells were treated with ACA, and total and exosomal protein isolation was carried out and analyzed. Considering the findings, our molecular dynamics simulation of the ACA-protein complexes, spanning 100 ns, conclusively demonstrated stable protein–ligand interactions. Additionally, ACA was determined to regulate LC3II, Beclin-1, p62, and Lamp2a protein levels and reduce amyloid-β and Alix protein levels. In conclusion, our study highlights the significant in vitro neuroprotective effect of ACA against Aβ toxicity through autophagy. Moving forward, future studies may seek to elucidate the specific neuroprotective, therapeutic effects and mechanisms of ACA via autophagy in in vivo models. Addressing the identified limitations and capitalizing on the outlined future prospects are essential steps towards harnessing the therapeutic potential of ACA in combating neurodegenerative diseases, offering renewed hope for patients and caregivers alike.

**Supplementary Information:**

The online version contains supplementary material available at 10.1007/s12035-025-04908-3.

## Introduction

Alzheimer’s disease (AD) is a progressive neurodegenerative disease characterized by memory loss and cognitive impairment [1]. In recent years, an instantaneous boost in the number of affected individuals has been seen. It is estimated that 107 million people worldwide will be affected by 2050 [2]. At the neuropathological level, AD is distinguished by the deposition of amyloid beta (Aβ) fibrils outside neurons and the hyperphosphorylation of tau protein within neurons [3].

Autophagy is a process in which the cytoplasm containing excess or damaged organelles, misfolded or aggregated proteins, and intracellular pathogens are enclosed in a double-membrane vesicle, delivered to the lysosome for degradation and eventual recycling [4]. Autophagy is a molecular mechanism involved in cell growth, survival, development, and death. Autophagy pathway dysregulation is linked to the pathophysiology of many diseases, including neurodegeneration. To maintain proper cellular function, the cell’s autophagy level must be regulated appropriately [5]. Since autophagic dysfunctions such as immature autophagic vacuoles, inhibition of the fusion of the autophagosome with a lysosome, decreased lysosomal acidification, and inability to destroy autophagosomes can cause neurodegeneration, it is imperative to investigate the place of autophagy in the pathobiology of AD [6]. However, autophagy pathway irregularities in neurons may cause the spread of neurodegenerative diseases by increasing exosome-mediated toxic protein transport [7]. Therefore, understanding the autophagy-exosome relationship and manipulating their communication is a therapeutic strategy [7, 8].

Three main types of autophagy are defined: macroautophagy, microautophagy, and chaperone-mediated autophagy [9]**.** The process of macroautophagy begins with the entrapment of material within a double-membrane vesicle called an autophagosome. First, unconjugated LC3I is converted into LC3II conjugated with phosphatidylethanolamine, which is associated with the autophagosome membrane [10]. Therefore, the ratio of LC3II to LC3I is considered an indicator of autophagy triggering. Beclin-1 is an autophagy-specific protein that regulates autophagosome formation. Beclin-1 induces or suppresses the autophagy pathway by interacting with other proteins. Interaction with PI3K causes increased autophagy, whereas interaction with Bcl-2 causes inhibition of autophagy [11]. Studies on autophagy and neurodegeneration have shown that Beclin-1 level is closely related to neurodegeneration, decreasing in early stage AD. Therefore, impaired Beclin-1 expression increases amyloid-β accumulation in AD. Impaired Beclin-1 expression has been reported to increase amyloid-β accumulation in AD [12]. In the final stage of the macroautophagy process, the autophagosome fuses with the lysosome to form the autolysosome together with lysosomal membrane proteins, vacuolar proton pumps and acid hydrolases. The process of macroautophagy ends with the breakdown of the material [13]. Lamp2a is one of the lysosomal membrane proteins that maintains lysosomal stability and participates in autophagy. Autophagic vacuole accumulation has been observed in various tissues of Lamp2-deficient mice. Lamp2a is the receptor for chaperone-mediated autophagy, a lysosomal proteolytic process that is activated during starvation and eliminates damaged cellular proteins [14]. Apart from the three main types of autophagy, types of autophagy that occur selectively under certain conditions have also been described. One of the best characterized proteins of selective autophagy is p62 [9]. p62 functions as an autophagy receptor in the clearance of unwanted protein molecules and aggregates. By directly interacting with LC3, it is included in the autophagosome and ensures the degradation of protein aggregates in lysosomes [9, 15]. Therefore, p62 accumulation is considered an important marker in determining the level of defective or impaired autophagy [16].

Acacetin (ACA) is a molecule that has been studied in different pathologies worldwide due to its various biological activities, including neuroprotective, cardioprotective, anti-aging, anti-cancer, anti-inflammatory, and anti-microbial properties. It is predicted to be a potential therapeutic compound for treating AD [17–21]. However, more molecular studies are needed to reveal the therapeutic effect of ACA. Considering the autophagy pathway irregularities in AD and the spread of toxic proteins into cells via exosome-mediated transport due to autophagy dysregulation, it is crucial to investigate the effects of ACA on the autophagy mechanism and autophagy-related exosome release in AD. Remarkably, autophagy pathway dysregulation is linked to the pathophysiology of AD. ACA, a natural flavonoid compound, has emerged as a potential therapeutic agent due to its neuroprotective properties. Considering the impairment of autophagy pathway in AD it is important to investigate the effects of ACA on the autophagy mechanism. Also, one of primary reasons for spread of toxic proteins into cells via exosome-mediated transport is dysregulation of autophagy. This knowledge highlights the cross-talk between autophagy and exosome biogenesis. At this point, autophagy-related exosome release in AD is an area of interest.

**Fig. 1:**
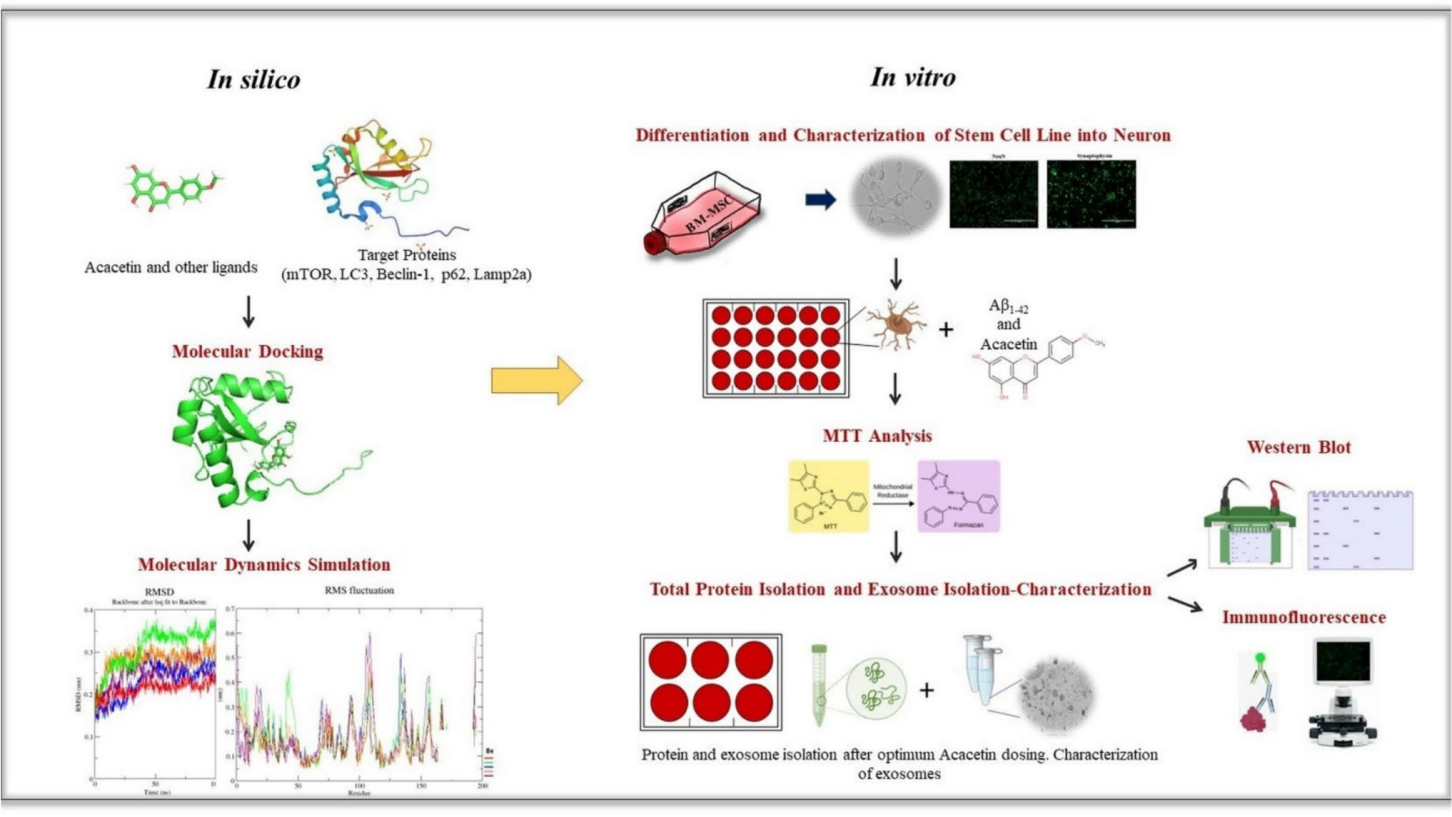
The pipeline showcases an in vitro characterization of autophagy target proteins following the in silico calculation

Our aim in the current study was to reveal the relation between exosome release and autophagy by examining the expression levels of proteins involved in the autophagy pathway. This approach provided us with information to underline the neuroprotective effects of ACA. For this purpose, we conducted an in vitro study besides in silico practices (see Fig. 1). Our study showed that ACA treatment can reduce the level of Aβ_1–42_, via regulating autophagy pathway and exosome release dynamics. Regarding its therapeutic role, our findings suggested that ACA may have neuroprotective effects that could be beneficial for development of ACA-based therapies in AD.

## Materials and Methods

### Pre-preparation Step for Molecular Modeling

Molecular docking was executed to determine the binding mode of ACA to autophagy pathway related to target proteins (mTOR PDB ID: 4DRJ, LC3 PDB ID: 5DCN, Beclin-1 PDB ID: 6HOI, p62 PDB ID: 5YP7, Lamp2a PDB ID: 2MOM). Before commencing the protein’s docking procedure, meticulous preparations are required with its crystal structures, which entail tasks beyond the scope of X-ray crystal structure refinement. These tasks necessitate the optimization of hydrogen bonds, the elimination of atomic clashes, and the execution of other operations. To acquire the X-ray crystallography structure for the target protein, the following steps were meticulously carried out: (I) Extraction of water and ligands, (II) Addition of polar hydrogen bonds, (III) Energy minimization of all 3D proteins was executed faultlessly using Chimera 1.14 [22].

### Ligan-Based Similarity

To evaluate the conformation of residues that ACA binds to target proteins and to increase the number of repetitions of molecular dynamics simulations, biocomponents with high structural similarity to ACA were identified (Apigenin, Diosmetin, Genkwanin, Pectolinarigenin) using the SwissSimilarity web tool (http://www.swisssimilarity.ch/). The interaction network of these detected biocomponents and ACA with proteins was revealed using the STITCH database (http://stitch.embl.de/).

### Molecular Docking

Molecular docking was conducted employing the fully automated Achilles Blind Docking Server (https://bio-hpc.eu/software/blind-docking-server/). Visualization of the docking complexes was achieved with the BIOVIA Discovery Studio software by taking the best poses of protein–ligand interactions [23].

### Molecular Dynamics Simulation

A molecular dynamics simulation was performed to test the structural stabilization of protein–ligand interactions and examine their interactions at the atomic level. For this purpose, after the molecular docking, the protein–ligand interaction with the highest binding affinity (kcal/mol) was selected, and the GROMACS package program was used via the WebGro server (https://simlab.uams.edu). The simulation was sequentially processed in four main steps: Step 1, The topology file of protein–ligand complexes was created with the PRODRG service tool (http://davapc1.bioch.dundee.ac.uk/cgi-bin/prodrg), which was employed to generate ligand topology for preprocessing, which includes protein and ligand topology generation. The SPC (simple point charge) as water model and triclinic box type opted [24]. Depending on the total charges, the system was neutralized by providing sufficient sodium and chloride ions (0.15 M) salt. Step 2, for energy minimization (EM), EM Integrator is set up as the steepest descent algorithm with 5000 steps. In step 3, the simulation system was equilibrated using the NVT/NPT method. The system was first equilibrated at constant volume and temperature (NVT) with 300 K and pressure as 1.0 bar, followed by an additional equilibration step at constant pressure and temperature (NPT) and an approximate number of frames per simulation as 1000. Step 4, the simulation time was carried out with a leap-frog MD integrator with a duration of 100 ns. Root mean square deviation (RMSD) and root mean square fluctuation (RMSF) measurements were taken [25]. Additionally, the plot of solvent accessible surface area (SASA), radius of gyration (Rg), and percentage of hydrogen bond occupancy (protein–ligand) were evaluated.

### Cell Culture and Cell Differentiation

A bone-derived human mesenchymal stem cell (BMMSC) line was obtained from ATCC and differentiated into neuron cells. BMMSC were planted in a 25 cm^2^ culture dish with Dulbecco’s Modified Eagle Medium (DMEM; DMEM-LPA, Capricorn) containing 10% Fetal Bovine Serum (FBS; FBS- 11 A, Capricorn), 1% penicillin/streptomycin (15140122, Gibco) and propagated in an incubator containing 37 °C, 5% CO_2_ and 95% humidity. The medium available for the neuronal differentiation process was replaced with serum-free neurobasal medium containing 20 ng/ml human epidermal growth factor (hEGF; 585,506, Biolegend), 40 ng/ml basic fibroblast growth factor (bFGF; 571502, Biolegend), 10 ng/ml fibroblast growth factor- 8 (FGF- 8; 773502, Biolegend), 10 ng/ml human brain-derived neurotrophic factor (BDNF; 788,902, Biolegend), 40 ng/ml beta nerve growth factor (β-NGF; 788504, Biolegend), 0.254 mM dibutyryl cyclic AMP (dbcAMP; A15914, Adooq Bioscience), 0.5 mM IBMX (3-isobutyl- 1-methylxanthine; 15,879, Sigma), 2% B27 (17404044, Thermo Fisher Scientific), 1% penicillin/streptomycin (15140,122, Gibco, 1% L-glutamine (2 mM) (25034–024, Gibco). During the differentiation process, which continued for 6 days, the neuronal induction medium was replaced with a new one every 48 h [26]. Neuron cells generated by inducing mesenchymal stem cells were analyzed with NeuN (24307, Cell Signaling Technology) and synaptophysin (837104, Biolegend) for neuron-specific phenotypic features.

### Amyloid Beta Peptide-Induced Toxicity Model

BMMSCs were reproduced at a density of 6 × 10^4^ cells/well in a 24-well culture dish and differentiated into neuron cells. On the 4 th day of differentiation, cells were treated with NH_4_OH-soluble Aβ_1–42_ peptide (A14075 - 1, Adooq Bioscience) at concentrations of 1.25, 2.5, 5, 10, and 20 μM. It was incubated for 48 h. At the end of the incubation, MTT (3-(4,5-dimethylthiazol- 2-yl)− 2,5-diphenyltetrazolium bromide) (StKa00268, Clinisciences) test was performed. Thus, the appropriate concentration to create Aβ toxicity was determined.

### Acacetin Exposure and MTT Analysis

The ACA (00017, Sigma-Aldrich) concentrations (0 μM, 25 μM, 50 μM, 75 μM, 100 μM, 125 μM, 150 μM, 200 μM, 250 μM) were prepared in DMSO (Dimethyl sulphoxide) and applied for 24 h to the Aβ peptide-induced toxicity model cells. For MTT, cells were grown at 1 × 10^4^ cells/well in 96-well culture dishes, differentiated into neuron cells, and exposed. At the end of the exposure period, 10 µl, 5 mg/ml MTT solution was added to the culture medium and incubated at 37 °C for 4 h. Then, MTT solution was poured and 100 µl DMSO was added to the cells and incubated for 5 min at room temperature. The color change was recorded at 492 nm in a spectrophotometer, which is a colorimetric reader (Allsheng AMR- 100) [27]. Thus, the optimum ACA concentration effective on Aβ peptide-induced toxicity model cells was determined.

### Experimental Groups

All the experiments were carried out under stable laboratory conditions. Experimental groups were defined as; control, vehicle, Aβ_1–42_, Aβ_1–42_ + 25 μM ACA, and Aβ_1–42_ + 50 μM ACA. The convenient dosage of Aβ and ACA was determined by MTT assays. Control cells had no treatment, while vehicle cells were treated with dissolvers of Aβ (NH_4_OH) and ACA (DMSO). The group of cells with solely Aβ exposure were also treated with the given dissolvers. Cells were seeded and differentiated in 24-well plates for molecular studies. Wells were assigned for the experimental groups in a mixed way by random selection but side by side. At least 3 replicates were implemented for Western blot, immunoprecipitation, and immunofluorescence experiments.

### Exosome Isolation

Cells were multiplied and differentiated at 1.5 × 10^5^ cells/well in 6-well culture dishes. Then, the Aβ peptide-induced toxicity model was formed and incubated at optimum ACA capacity and time. Cell medium was collected and exosomes were isolated according to the instructions of the Cell Culture Media Exosome Purification Mini Kit (60,400, Norgen Biotek Corp.). Briefly, ExoC buffer and Slurry E were added to the cell medium. Vortexing and centrifugation were performed. ExoR buffer was added onto the pellet. Vortex and centrifuge were performed. The supernatant was transferred to a mini-filter spin column and centrifuged. Exosomes were filtered from the column, collected in the tube and stored at −80 °C. Exosomes were characterized with TEM and immunoprecipitated [28].

### Transmission Electron Microscopy

Exosomes were characterized by TEM (Carl Zeiss Microscopy) at Istanbul Medipol University, Research Institute for Health Sciences and Technologies (SABITA). Briefly, exosome samples were fixed with 2% paraformaldehyde, placed on formvar carbon-coated EM grids, and allowed to adsorb for 20 min at room temperature. The sample was washed 5 times with PBS for 2 min. The grid was incubated on uranylacetate-oxalate droplets for 5 min. The grid was then embedded in a 1:9 mixture of 4% uranyl acetate and 2% methylcellulose for 5 min. The grid was removed and excess liquid was dried with filter paper. After the drying process, images were examined on TEM [29].

### Exosome Immunoprecipitation

Exosome immunoprecipitation was carried out in the same manner as in our prior investigation, following the manufacturer's indirect immunoprecipitation methodology [28]. In brief, 100 µl of exosome was combined with 2 µl of Alix antibody (2171 s, Cell Signaling) and incubated overnight at 4^◦^C with constant mixing. The next day, 50 µl of Pure Proteome Protein G Magnetic Beads (LSKMAGG02, Millipore) were washed. The magnetic beads were incubated at room temperature for 10 min with continuous mixing with a pre-formed exosome-antibody combination. The exosome-antibody mixture was then removed, and the beads were washed. NuPAGE LDS Sample Buffer (NP0007, Thermo Scientific) and NuPAGE Sample Reducing Agent (NP0009, Thermo Scientific) were used to denature immunoprecipitated exosomes [28].

### Total Protein Isolation and Western Blot

Cells were multiplied and differentiated at 1.5 × 10^5^ cells/well in 6-well culture dishes. Then, the Aβ peptide-induced toxicity model was created and the cells were incubated at the optimum ACA concentration and time. Cells were collected from culture dishes and protein isolation was performed using lysis buffer containing 1% protease inhibitor cocktail. The ice-keeping and centrifugation phase was carried out. Protein concentration (mg/mL) was measured at an OD value of 260/280 from the supernatant obtained after centrifugation (diluted 1/20) with the DeNovix DS- 11 FX NanoDrop device. For each group, 40 μg of protein was denatured. Proteins were loaded onto NuPAGE 4–12% Bis–Tris Gel (NP0321BOX, Thermo Scientific) and subjected to electrophoresis. Samples in the gel were transferred to PVDF membrane (IB24001, Thermo Scientific). The membrane was incubated with 5% skim milk powder prepared in TBS-T (Tris-buffered saline (TBS) containing Tween- 20) for 1 h. Then, primary antibodies: LC3 A/B (1:500, 4108, Cell Signaling Technology), Beclin-1 (1:1000, 3495, Cell Signaling Technology), p62 (1:1000, E-AB- 62289, Elabscience), Lamp2a (1:1000, Ab18528, Abcam), Amyloid-β (1:1000, 8243, Cell Signaling Technology), Alix (1:1000, 2171S, Cell Signaling Technology), β-Actin (1:1000, 3700S, Cell Signaling Technology) were incubated at 4 °C for 16 h. The next day, after several washes with TBS-T, the secondary antibody (HRP Goat anti-mouse IgG, 405306, Biolegend and HRP Donkey anti-rabbit IgG, 406401, Biolegend) was incubated for 1 h at room temperature and the immune complexes were visualized by chemiluminescence method. The optical densities of the bands belonging to the investigated proteins and the β-Actin as a loading control were analyzed by Image J software (NIH, Washington, USA). Optical densities of the immunoreactive protein bands were normalized by comparing them to the corresponding re-probed β-Actin bands [28]

### Immunofluorescence

Intracellular localization of LC3, p62, and Lamp2a together with amyloid-β was examined under the Evos FL microscope. Briefly, cells were multiplied and differentiated in 24-well culture dishes on coverslips at 5 × 10^4^ cells/well. Then, the Aβ peptide-induced toxicity model was created, and the cells were incubated at the optimum ACA concentration and time. Cells were fixed with 4% paraformaldehyde (100% cold methanol was used for LC3 antibody) for 15 min. Blocking was done with PBS containing 1% bovine serum albumin for 1 h. Cells were incubated with primary antibodies (1:100 ratio) for 16 h at 4 °C. Cells were incubated with secondary antibody (1:300) (Alexa fluor 488 goat anti-mouse, A11001, Thermo Fisher Scientific and Alexa fluor 633 goat anti rabbit, A21070, Thermo Fisher Scientific) for 1 h at room temperature. Samples were counterstained with 4’,6-diamidino- 2-phenylindole (DAPI; AB- 104139, Abcam), and images were examined under a fluorescence microscope. Counting was done from 15 cell images taken at 20 × using the cell counting plug-in in the Image J program. It was analyzed semiquantitatively through a modified H-SCORE analysis that assigns numerical scores ranging from 0 to 300 depending on staining intensity [30].

### Statistical Analysis

Analysis results were obtained using GraphPad Prism Version 5.01 (GraphPad Software Inc., USA) program. All graphs were created with GraphPad Prism Version 5.01. Normal distribution of data within the group was determined by the Shapiro–Wilk test. One-way analysis of variance (ANOVA) by Tukey was used to determine significant differences between groups. Mean ± standard deviation (SD) was calculated for the experimental data. Each test group was compared with the appropriate control group, and *p* < 0.05, *p* < 0.01, *p* < 0.001 values were considered statistically significant.

## Results

### Molecular Docking

The binding energies between ACA, Apigenin, Diosmetin, Genkwanin, and Pectolinarigenin ligands and mTOR, LC3, Beclin-1, p62, and Lamp2a target proteins were relatively high (Table 1). 2D and 3D images of the mTOR and ACA binding sites were taken. Additionally, protein–ligand binding sites were identified (Supplementary file).

**Table 1:** The binding energy of acacetin and other biocomponents with autophagic pathway proteins (kcal/mol)

	mTOR	LC3	Beclin- 1	p62	Lamp2a
Acacetin	− 7,60	− 6,80	− 7,30	− 6,80	− 5,50
Apigenin	− 7,80	− 7,00	− 7,50	− 6,80	− 5,70
Diosmetin	− 7,70	− 7,00	− 7,10	− 6,80	− 5,50
Genkwanin	− 7,20	− 6,90	− 7,20	− 6,50	− 5,60
Pectolinarigenin	− 6,80	− 6,60	− 6,50	− 6,60	− 5,30

### Molecular Dynamics Simulation

**Fig. 2:**
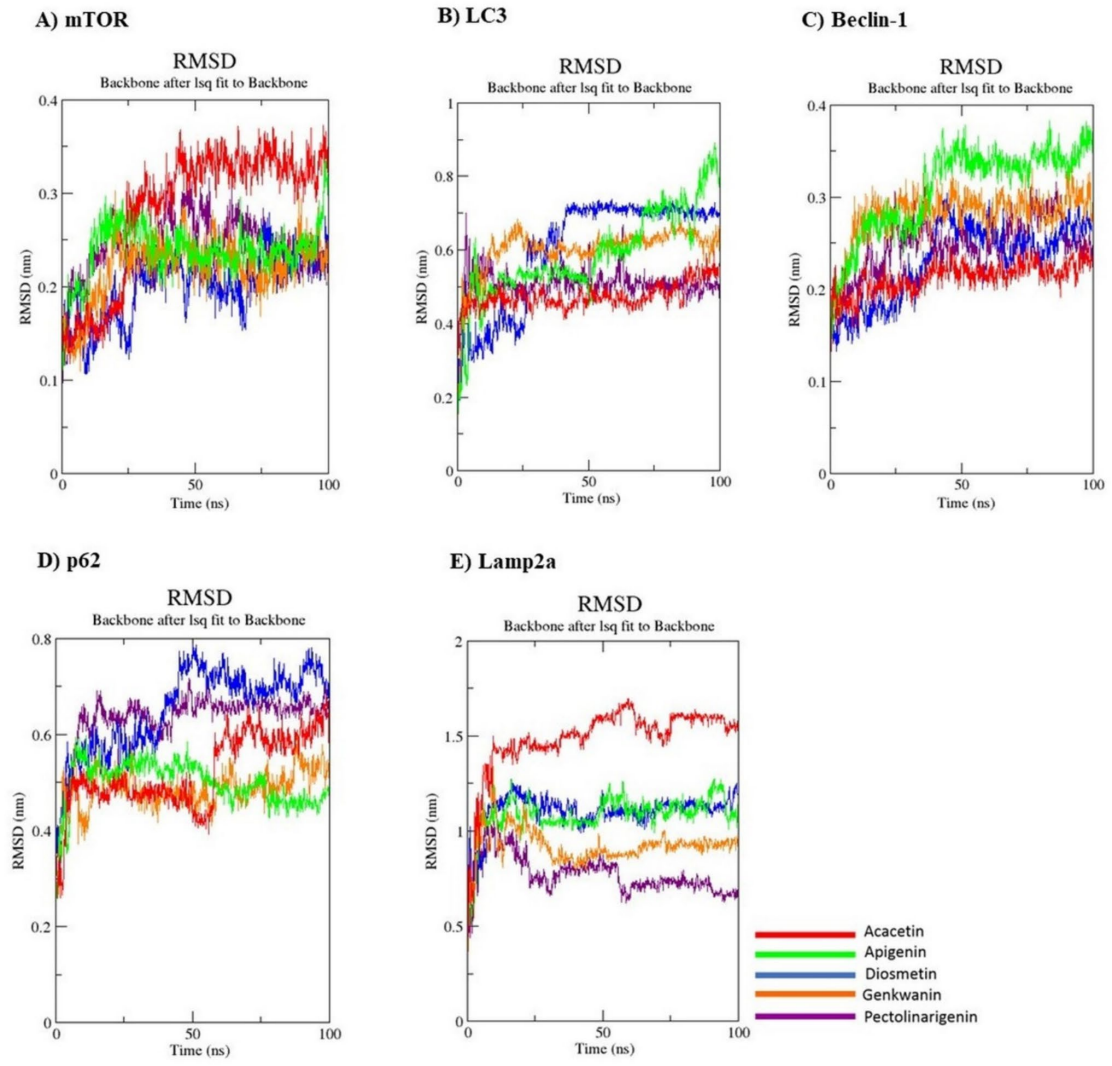
Molecular dynamics simulation with WebGro. Protein–ligand RMSD plots were evaluated over a 100 ns period. **A** mTOR and ligands, **B** LC3 and ligands, **C** Beclin-1 and ligands, **D** p62 and ligands, **E** Lamp2a and ligands

The RMSD plot provides basic information about time-dependent shifts and deviations of protein and ligand. This chart accepts values ranging from 0.1 to 0.3 nm for small proteins. Although some deviations were observed in the 100 ns RMSD graphs, except for the p62 protein graph, the complex values formed by ACA for all proteins did not show shifts greater than 0.1–0.3 nm. This indicates that ACA interactions with target autophagic proteins are stable (Fig. 2).

**Fig. 3:**
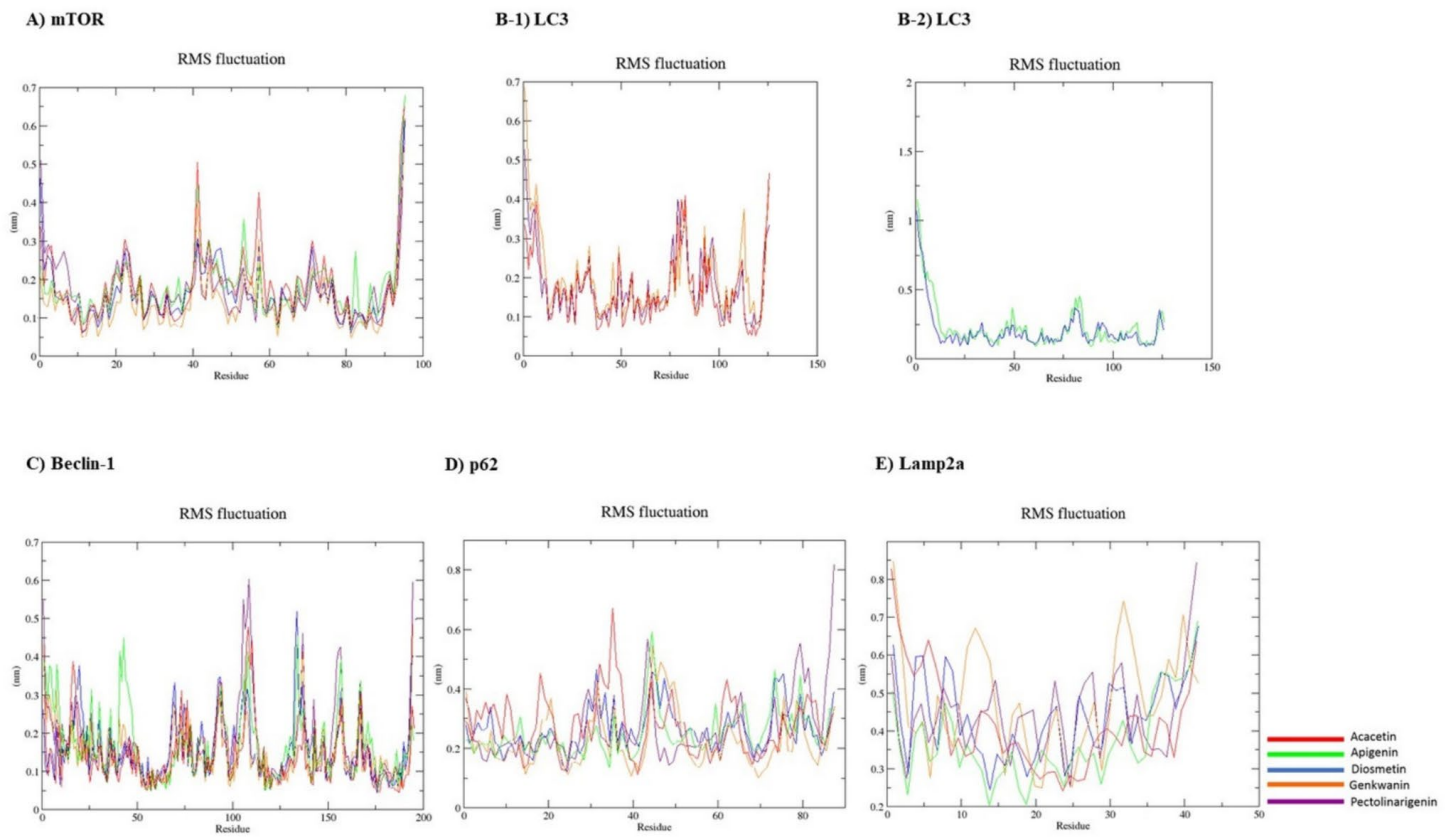
Molecular dynamics simulation with WebGro. Protein–ligand RMSF plots were evaluated over a 100 ns period. **A** mTOR protein and ligands, **B**− 1, **B**− 2 LC3 protein and ligands, **C** Beclin-1 protein and ligands, **D** p62 protein and ligands, **E** Lamp2a protein and ligands

The RMSF graph provides information about the fluctuation and conformational change of the protein. During the simulation, the parts of the protein that fluctuate the most can be observed as peaks. The fact that the fluctuations are very close in the RMSF graph shows that the stability of the complexes is good. In Fig. 2, the residue dynamics of proteins and ACA show fluctuations mostly at 0.4–0.5 nm and sometimes even higher fluctuations at the C-terminus. This information showed that the mean RMSF values exhibited similar patterns for all ligands, although p62 and Lamp2a were also relatively different (Fig. 3).

### Characterization of Neuron Cells Generated by Induction of Mesenchymal Stem Cells

**Fig. 4:**
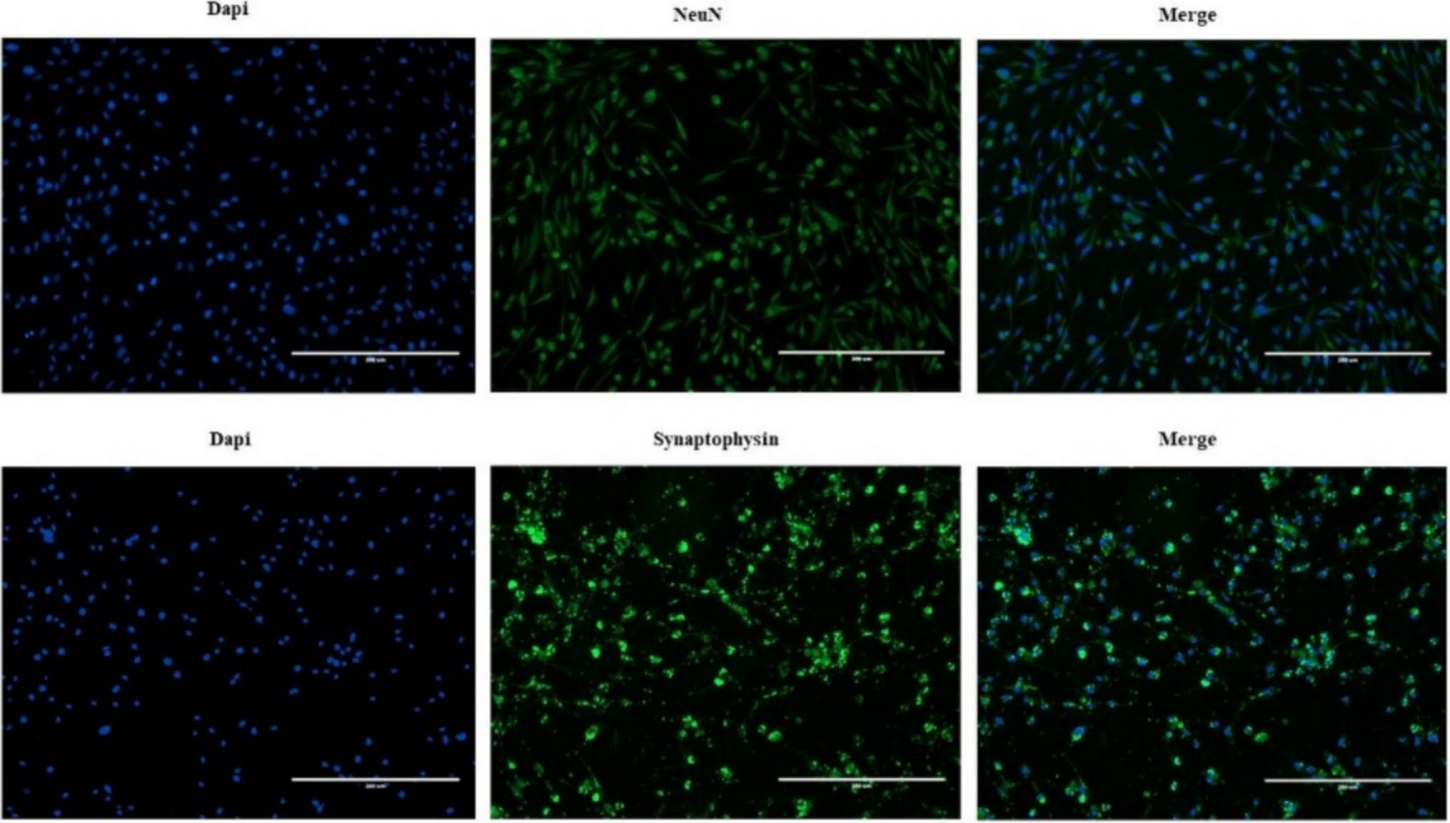
Characterization of neuron cells

Neuron cells created by differentiation of the mesenchymal stem cell line were stained with NeuN and synaptophysin antibodies for neuron-specific phenotypic features. NeuN and synaptophysin immunoreactivity were observed (Fig. 4).

### Effect of Amyloid Beta Toxicity on Cells

**Fig. 5:**
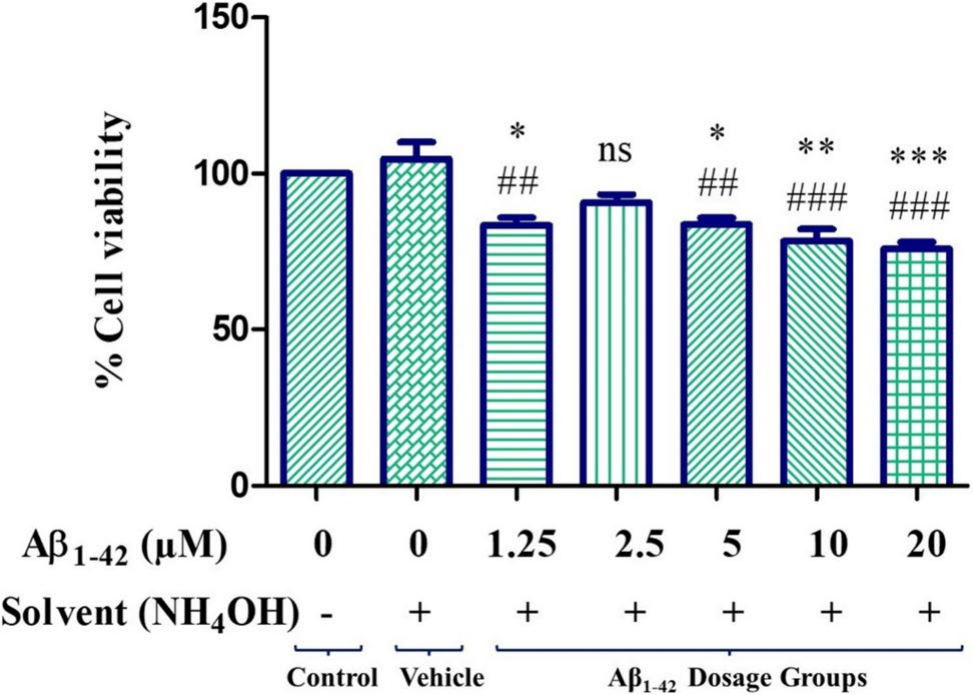
MTT analysis showing the effect of Aβ_1–42_ peptide on neuron cell viability. According to the results of statistical analysis performed with one-way ANOVA, Tukey’s multiple comparison test, statistical significance is ^*^p < 0.05, ^**^p < 0.01, ^***^p < 0.001 according to the control, ^##^*p* < 0.01, ^###^*p* < 0.001 according to the vehicle. ns means not significant statistically

MTT was performed to detect the toxic Aβ_1–42_ concentration on neuron cells, which was used to create an Aβ peptide-induced toxicity model. It was observed that 1.25, 5, 10, and 20 µM concentrations of Aβ_1–42_ peptide significantly suppressed cell viability compared to both the control and vehicle groups (Fig. 5). Considering the concentration values that suppress cell viability, the appropriate concentration to create the toxicity model was determined as 10 µM with ^**^p < 0.01 and ^*###*^*p < *0.001.

### Effect of Acacetin on Cells in Amyloid Beta Peptide-Induced Toxicity Models

**Fig. 6:**
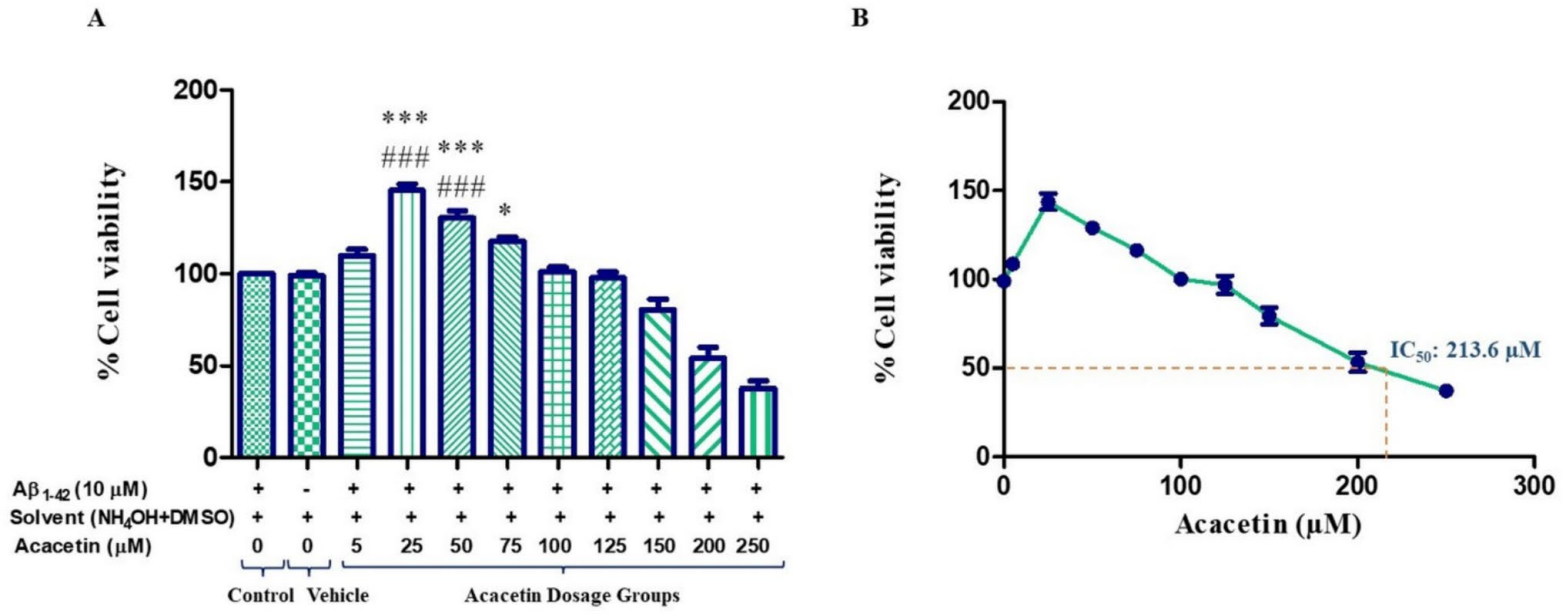
**A** MTT analysis showing the effect of acacetin on cell viability in Aβ peptide-induced toxicity model. According to the results of statistical analysis performed with one-way ANOVA and Tukey’s multiple comparison test, statistical significance is expressed as ^*^p < 0.05, ^***^p < 0.001 compared to the control, and ^###^*p* < 0.001 compared to the vehicle. **B** MTT analysis showing the dose-dependent change graph of acacetin in Aβ peptide-induced toxicity model. The calculated IC_50_ for acacetin is 213.6 µM

MTT was performed to determine the optimum ACA concentration on Aβ peptide-induced toxicity model cells. According to the findings, it was observed that 25 and 50 µM ACA concentrations significantly increased cell viability compared to both the control and vehicle groups. It was observed that 75 µM ACA concentration significantly increased cell viability only compared to the control group (Fig. 6).

### Characterization of Exosomes

**Fig. 7:**
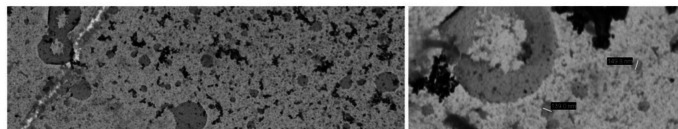
Morphological images of exosomes obtained by TEM

Exosomes obtained from the medium of neuron cells were characterized by TEM. Typical morphology of exosomes with different sizes was observed (Fig. 7).

### Western Blot Results of Autophagy Proteins

**Fig. 8:**
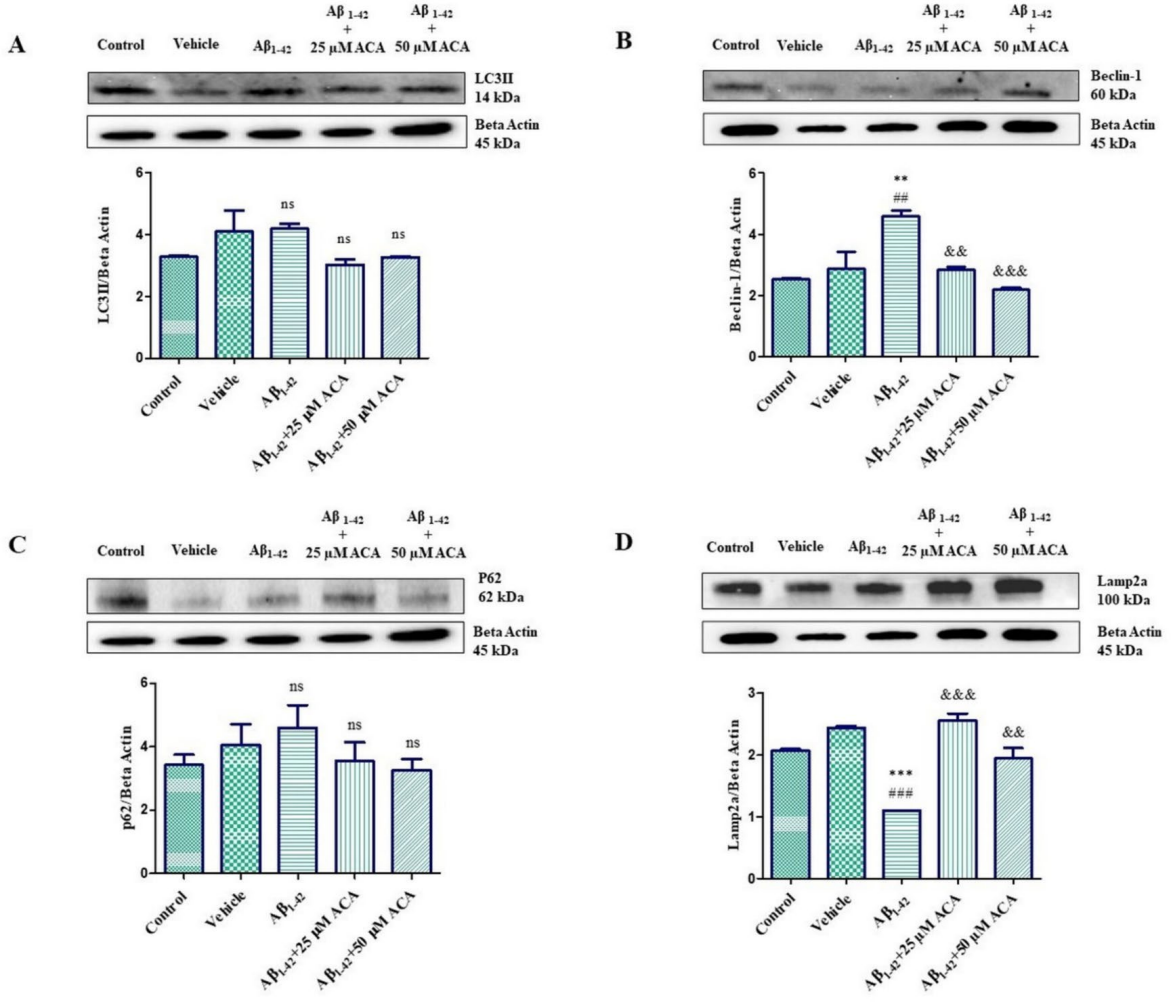
**A** Western blot analysis showing the change in LC3II/Beta Actin protein level. **B** Western blot analysis showing the change in Beclin-1/Beta Actin protein level. **C** Western blot analysis showing the change in p62/Beta Actin protein level. **D** Western blot analysis showing the change in Lamp2a/Beta Actin protein level. According to the results of statistical analysis performed with one-way ANOVA and Tukey’s multiple comparison test, statistical significance is expressed as ^**^p < 0.01, ^***^p < 0.001 compared to the control, ^##^*p* < 0.01, ^###^*p* < 0.001 according to the vehicle, ^&&^*p* < 0.01, ^&&&^*p* < 0.001 according to the Aβ_1–42_ group. ns means not significant statistically. ACA; acacetin

According to the findings, it was observed that the LC3II protein, which participates in the autophagosome structure, which is a marker of the induction of autophagy, increased in the Aβ_1–42_ group compared to the control and vehicle groups, and decreased in the Aβ_1–42_ + 25 µM and Aβ_1–42_ + 50 µM ACA groups. It was determined that Beclin-1 protein, which is a marker for the autophagosome initiation complex, increased significantly in the Aβ_1–42_ group compared to the control and vehicle groups and decreased significantly in the Aβ_1–42_ + 25 µM and Aβ_1–42_ + 50 µM ACA groups. It was observed that P62 protein, which participates in the autophagosome structure, increased in the Aβ_1–42_ group compared to the control and vehicle groups, and decreased in the Aβ_1–42_ + 25 µM and Aβ_1–42_ + 50 µM ACA groups. It was determined that the autophagolysosome structure and chaperone-mediated autophagy marker Lamp2a protein, unlike the others, significantly reduced in the Aβ_1–42_ group compared to the control and vehicle groups, and increased significantly in the Aβ_1–42_ + 25 µM and Aβ_1–42_ + 50 µM ACA groups (Fig. 8).

### Western Blot Result of Amyloid-β and Alix Protein

**Fig. 9:**
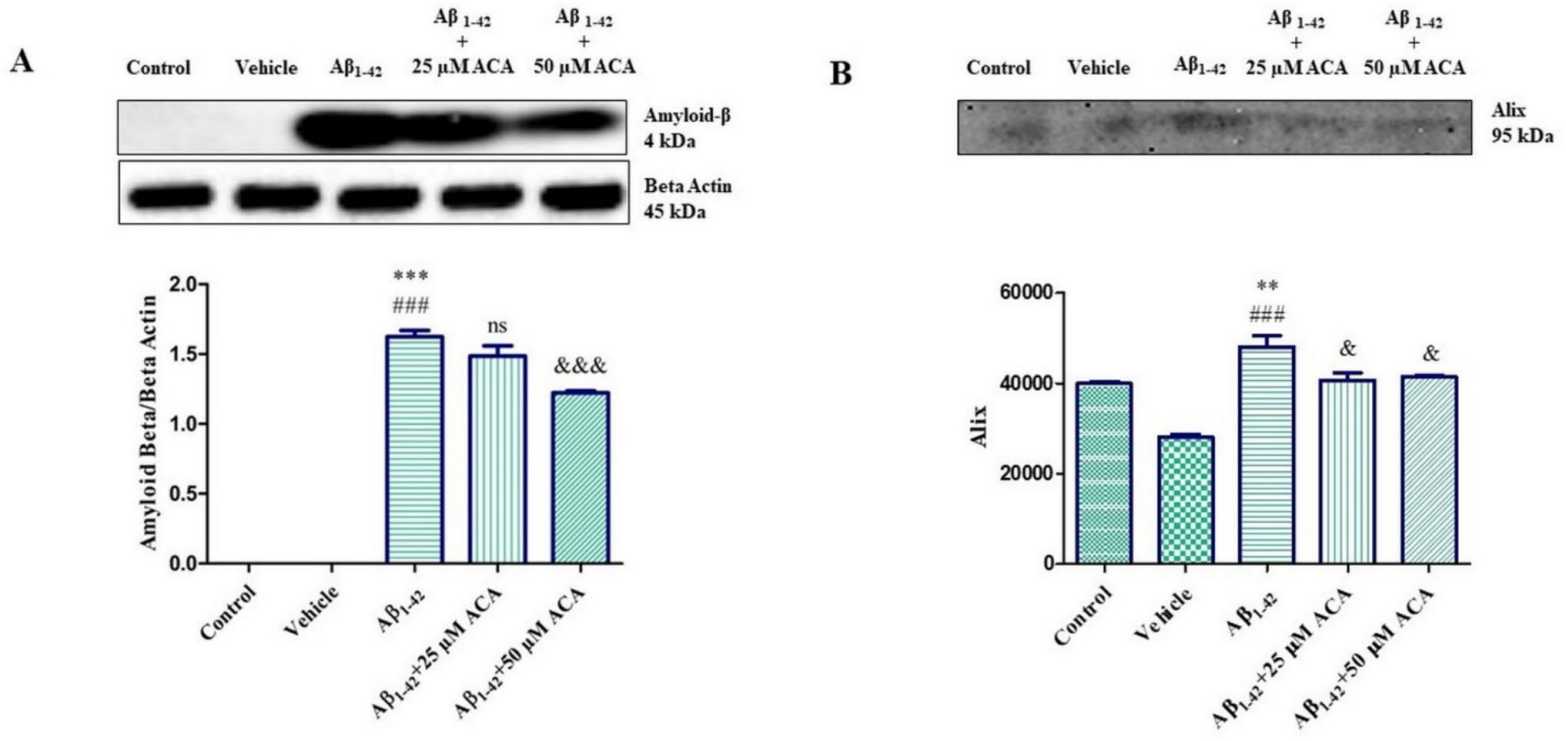
**A** Western blot analysis showing the change in amyloid-β protein level. **B** Western blot analysis showing the change in Alix protein level. According to the results of statistical analysis performed with one-way ANOVA and Tukey’s multiple comparison test, statistical significance is expressed as ^**^p < 0.01, ^***^p < 0.001 compared to the control, ^###^*p* < 0.001 according to the vehicle, and ^&^*p* < 0.05, ^&&&^*p* < 0.001 according to the Aβ_1–42_ group. ns means not significant statistically. ACA; acacetin

It was determined that amyloid beta protein increased significantly in the experimental model Aβ_1–42_ group compared to the control and vehicle groups. It was observed that amyloid beta protein decreased in the Aβ_1–42_ + 25 µM ACA group compared to the Aβ_1–42_ group, and it decreased significantly in the Aβ_1–42_ + 50 µM ACA group compared to the Aβ_1–42_ group (Fig. 9a). The change in the protein level of Alix, an exosome-associated protein, was determined from the isolated exosomes by western blot method. According to the findings, it was determined that Alix protein increased significantly in the Aβ_1–42_ group compared to both the control and vehicle groups. Alix protein level was observed to decrease significantly in the Aβ_1–42_ + 25 µM ACA and Aβ_1–42_ + 50 µM ACA groups (Fig. 9b).

### Immunofluorescence Results of Amyloid-β with Autophagy Proteins

**Fig. 10:**
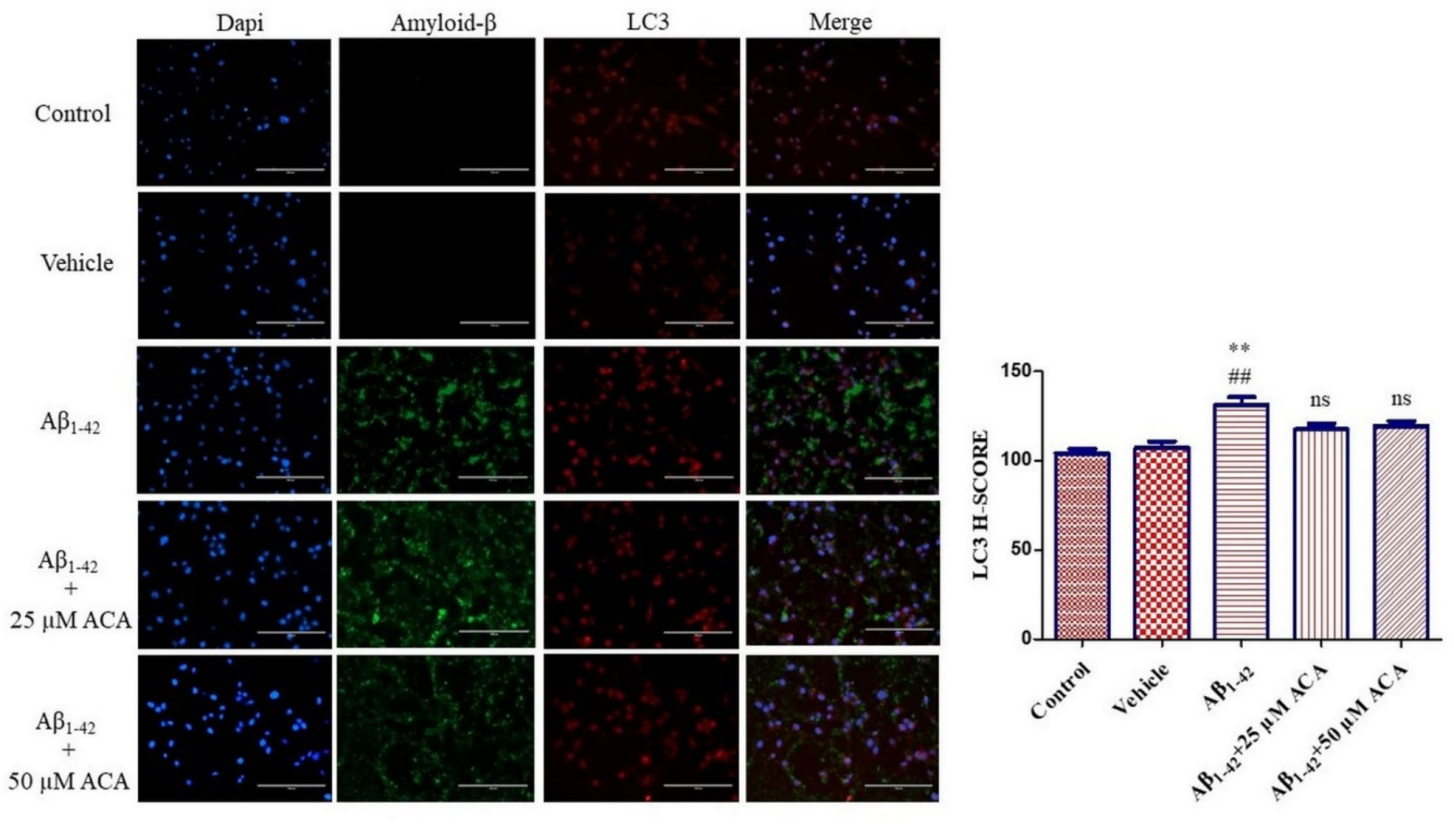
LC3 immunofluorescence analysis and amyloid beta localization. Neuron cells were treated with ACA. The nucleus was stained with dapi. Immunofluorescence analysis of LC3 was performed. Amyloid-β localization was detected. According to the results of statistical analysis performed with one-way ANOVA and Tukey’s multiple comparison test, the statistical significance is ^**^p < 0.01 compared to the control, ^##^*p* < 0.01 compared to the vehicle. ns means not significant statistically. ACA; acacetin

By performing immunofluorescence staining of the autophagosomal marker LC3 together with amyloid-β, the role of ACA in autophagosomal structure formation and the localization of amyloid-β plaques were determined. According to the findings, it was observed that autophagosome structure formation increased with a significant increase in LC3 level in the Aβ_1–42_ group compared to the control and vehicle groups. In the Aβ_1–42_ + 25 µM ACA and Aβ_1–42_ + 50 µM ACA applied groups, it was determined that the LC3 level decreased and approached the basal autophagosome level compared to the Aβ_1–42_ group. No accumulation of amyloid-β was observed in the control and vehicle groups, but its accumulation was observed around neuron cells in the other groups (Fig. 10).

**Fig. 11:**
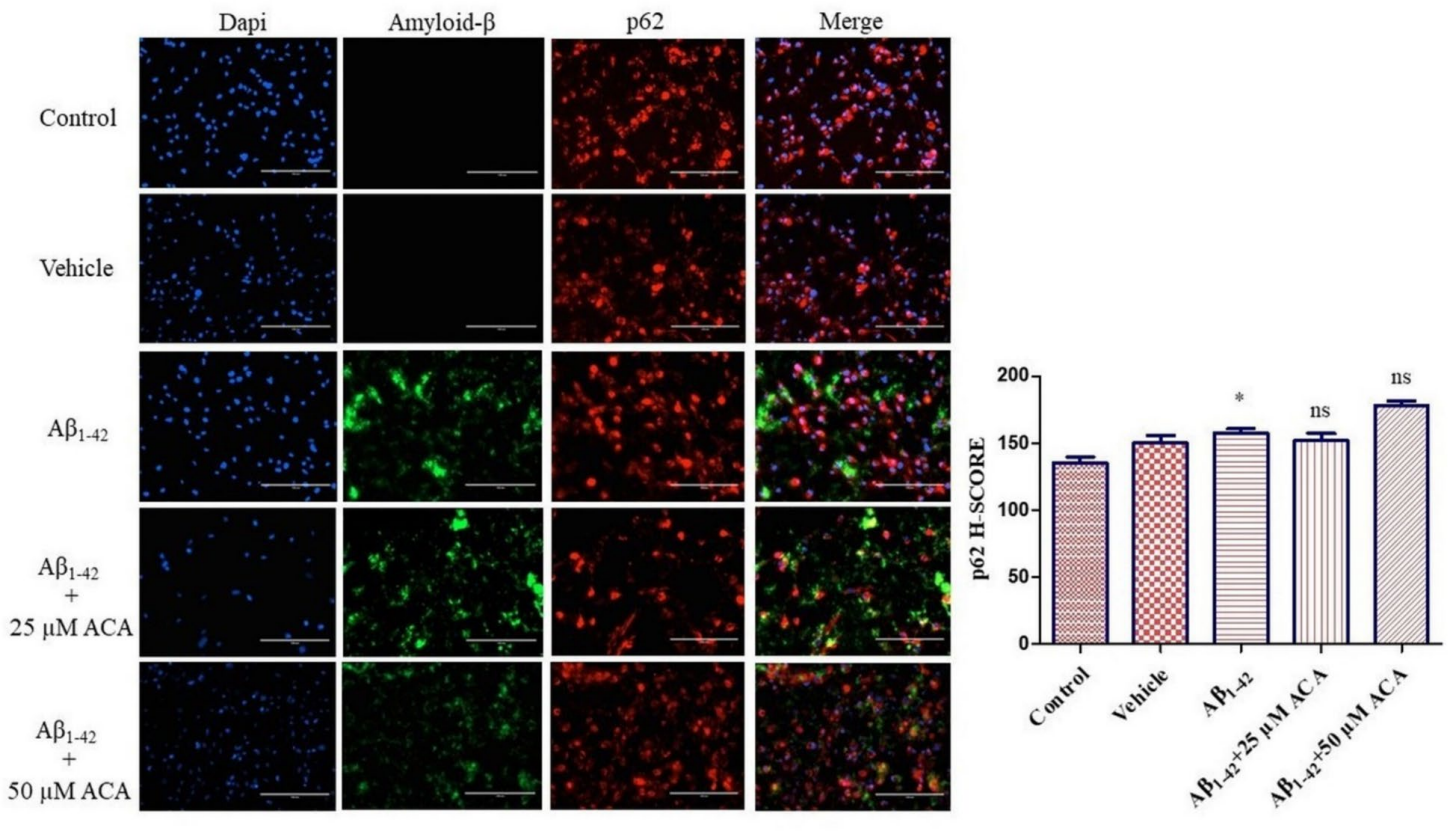
p62 immunofluorescence analysis and amyloid beta localization. Neuron cells were treated with ACA. The nucleus was stained with dapi. Immunofluorescence analysis of P62 was performed. Amyloid-β localization was detected. According to the results of statistical analysis performed with one-way ANOVA and Tukey’s multiple comparison test, statistical significance is ^*^p < 0.05 compared to the control. ns means not significant statistically. ACA; acacetin

By immunofluorescence staining of p62, which participates in the autophagosome structure, together with amyloid-β, the role of ACA in autophagic flow and the localization of amyloid-β plaques were determined. According to the findings, it was observed that autophagosome structure formation increased with a significant increase in p62 level in the Aβ_1–42_ group compared to the control group. It was observed that the p62 level decreased and approached the basal autophagy level in the Aβ_1–42_ + 25 µM ACA group compared to the Aβ_1–42_ group. An increase was detected in the Aβ_1–42_ + 50 µM ACA group compared to the Aβ_1–42_ group. Accumulation of amyloid-β was not observed in the control and vehicle groups, but its accumulation was observed around neuron cells in the other groups (Fig. 11).

**Fig. 12:**
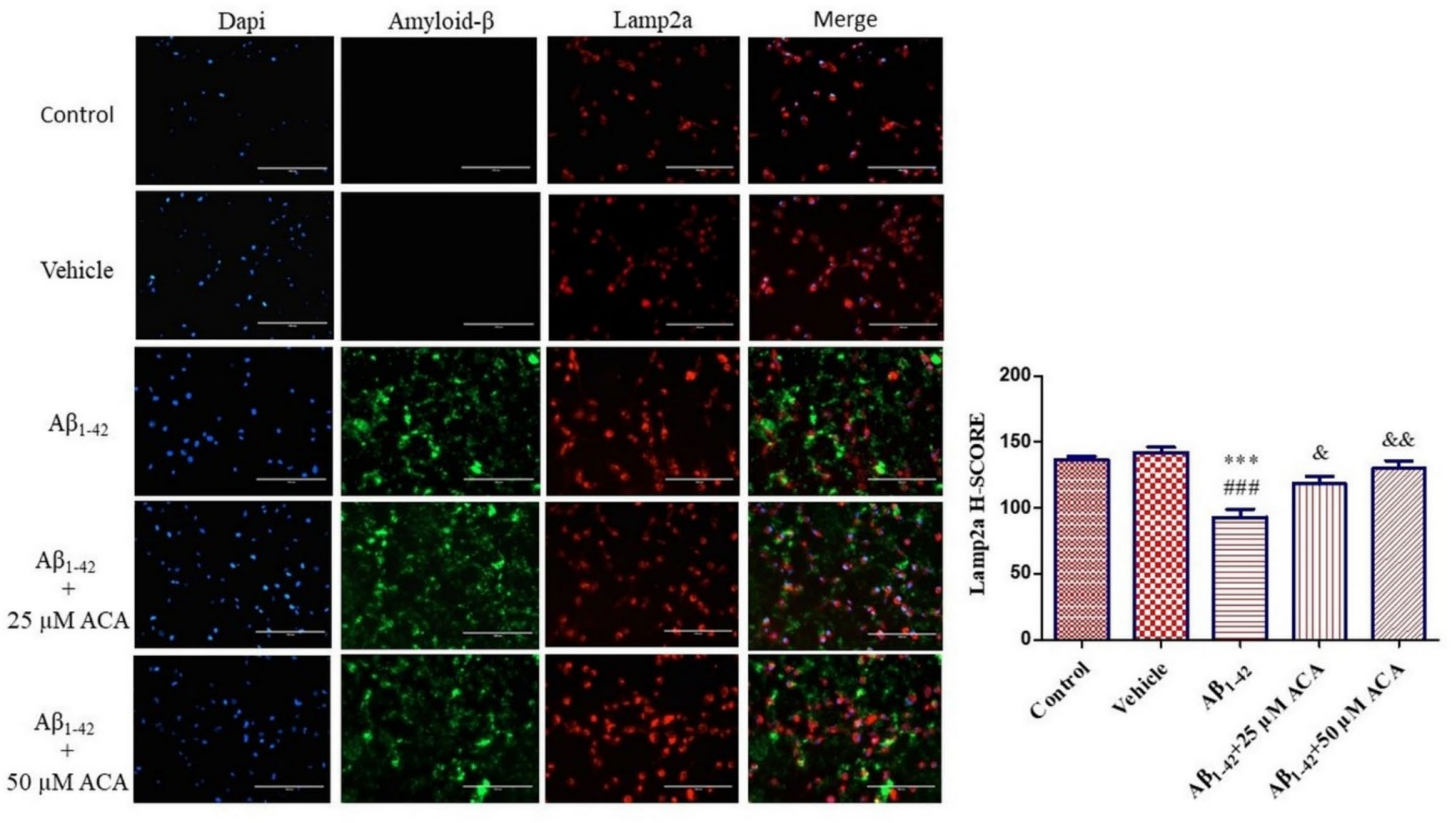
Lamp2a immunofluorescence analysis and amyloid beta localization. Neuron cells in Aβ peptide-induced toxicity model were treated with ACA. The nucleus was stained with dapi. Immunofluorescence analysis of Lamp2a was performed. Amyloid-β localization was detected. According to the results of statistical analysis performed with one-way ANOVA and Tukey’s multiple comparison test, statistical significance is expressed as ***p < 0.001 compared to the control, ^###^*p* < 0.001 according to the vehicle, ^&^*p* < 0.05, ^&&^*p* < 0.01 according to the Aβ_1–42_ group. ACA; acacetin

By immunofluorescence staining of Lamp2a, which participates in the autophagolysosome structure, together with amyloid-β, the role of ACA in autophagic flow and the localization of amyloid-β plaques were determined. According to the findings, it was observed that the Lamp2a level decreased significantly and the formation of autophagolysosome structure decreased in the Aβ_1–42_ group compared to both the control and vehicle groups. In the Aβ_1–42_ + 25 µM ACA and Aβ_1–42_ + 50 µM ACA applied groups, it was observed that the Lamp2a level increased significantly compared to the Aβ_1–42_ group, and the autophagolysosome structure approached the basal autophagic level. No accumulation of amyloid-β was observed in the control and vehicle groups, but its accumulation was observed around neuron cells in the other groups (Fig. 12).

## Discussion

Characterizing protein–ligand interactions using computer-based tools plays an important role in the drug discovery process [31]. Molecular docking and molecular dynamics are powerful tools used to investigate protein–ligand interactions. Molecular docking programs predict the binding pose and affinity of a protein–ligand complex. In contrast, molecular dynamics can be used to incorporate flexibility into docking calculations and learn more about the kinetics and stability of the protein–ligand bond [32]. Our study evaluated the binding affinity of autophagy pathway target proteins with ACA and detected relatively strong binding energies. Molecular dynamics simulations examined structural stabilization of affinity of autophagy pathway target proteins with ACA. The interactions were found to be stable.

 Autophagy is the major stress response to misfolded proteins, damaged organelles, and insoluble protein aggregates, and autophagy impairment is thought to play a role in neurodegenerative diseases [33]. LC3II is produced when autophagy is stimulated and transferred to newly formed autophagosomes. Therefore, its presence is indicative of the induction of autophagy [34]. In the LC3II protein analysis performed by immunoblotting and immunofluorescence, we found that the protein level increased in the Aβ_1–42_ toxicity group (at a statistically significant level in the immunofluorescence analysis). We observed that the protein increase decreased and reached the basal cell level in the Aβ_1–42_ + 25 µM ACA and Aβ_1–42_ + 50 µM ACA groups. LC3II levels have been reported to be significantly increased in the early stages of AD and to a greater extent in the mild/moderate stage of AD [34]. This information suggests that the autophagic pathway is triggered by the increase in LC3II level in response to increased stress in AD. Beclin-1 participates in the autophagosome structure by creating the isolation membrane, a double-membrane structure that will encapsulate the cytoplasmic material in the initial stage of autophagy [19]. In the Beclin-1 protein analysis, we found that the protein level increased in the Aβ_1–42_ toxicity group and increased at a statistically significant level. In the Aβ_1–42_ + 25 µM ACA and Aβ_1–42_ + 50 µM ACA applied groups, Beclin-1 protein level decreased significantly and reached the basal cell level. The increased protein level of Beclin 1, similar to the LC3II level in the Aβ_1–42_ toxicity group, indicates that autophagy is triggered. Contrary to our findings, it is reported in the literature that Beclin-1 expression levels decrease in AD brains in the early disease process [12, 35]. However, it has been reported that the decrease in Beclin-1 level facilitates the proteolytic cleavage of APP by reducing autophagic activity [35]. However, considering all the results, it seems that the increase in Beclin-1 protein level in our study does not necessarily indicate an increase in autophagy activity. p62 selectively enables proteins to bind to the autophagosome membrane and participate in the structure [15]. In western blot and immunofluorescence analysis of p62 protein, we found that the protein level increased in the Aβ_1–42_ toxicity group (at a statistically significant level compared to the control group according to immunofluorescence analysis). In immunoblotting analysis, we saw that the protein increase decreased and reached the basal cell level with the application of Aβ_1–42_ + 25 µM ACA and Aβ_1–42_ + 50 µM ACA. In immunofluorescence analysis, we found that while protein immunoreactivity decreased in the Aβ_1–42_ + 25 µM ACA group compared to the Aβ_1–42_ toxicity group, protein immunoreactivity increased in the Aβ_1–42_ + 50 µM ACA group, but this difference was not statistically significant. It has been reported that p62-mediated autophagy induction is also associated with increased levels of LC3II. However, it has been stated that removing the LC3-interacting region of p62 or blocking autophagy with a pharmacological inhibitor can prevent the decrease in amyloid-β [36]. Another study conducted by differentiating PC12 cells into neuronal cells showed that Aβ increased the expression and protein level of Beclin-1, LC3II, and p62 and caused autophagic flow defect with autophagosome accumulation [37]. Lamp2a mediates the transport and degradation of misfolded proteins through the Lamp2a channel to the lysosome [38]. In the Lamp2a protein analysis, we found that the protein level in the Aβ_1–42_ toxicity group decreased statistically significantly compared to the control and vehicle groups. We found that this protein level increased with ACA (Aβ_1–42_ + 25 µM and Aβ_1–42_ + 50 µM) application. It has been reported that the autophagy-lysosomal system is impaired in AD [36]. However, an examination of human Alzheimer’s brains showed autophagosome accumulation. It is thought that this situation may be caused by deficiencies in autophagy flow [34]. In a study examining the effects of ACA treatment in Hela cells, the status of autolysosomes was investigated with the Lamp1 protein, and it was observed that the fusion of autophagosomes and lysosomes increased [39]. Similarly, according to the data we obtained from western blot and immunofluorescence results, we determined that ACA regulates the autolysosome system, disrupted in AD, by increasing the Lamp2a level.

While the protein level and immunoreactivity of the amyloid-β protein, which we examined with western blot and immunofluorescence experiments, was not visible in the control and vehicle groups, it was visible in the groups treated with Aβ_1–42_ toxicity. These data show the accuracy of the experimental model we created on neuron cells. Despite the Aβ_1–42_ toxicity group, we found that the amyloid-β protein level decreased with the application of Aβ_1–42_ + 25 µM ACA, and the protein level decreased at a statistically significant level with the application of Aβ_1–42_ + 50 µM ACA. Similarly, in a study conducted in a Drosophila Alzheimer’s model, it was reported that ACA application reduced the amyloid-β protein level [40]. Our findings are consistent with the reducing effect of ACA on amyloid-β.

In the literature, exosome-associated proteins flotillin-1 and Alix proteins have been examined in exosomes containing amyloid plaques, and it has been reported that exosomal Aβ peptide may contribute to the formation of amyloid plaques in the extracellular space. However, it has been shown that the exosomal protein Alix is enriched around amyloid-β plaques, which may contribute to the formation of amyloid-β plaques associated with exosomes [41]. A study conducted in monkeys and APP transgenic mice demonstrated the presence of exosome-associated amyloid-β in the cerebrospinal fluid [42]. In another study, it was stated that exosomes isolated from AD patients contained amyloid-β, and using SH-SY5Y cells, it was reported that amyloid-β spread through exosomes and that in vitro toxicity could be reduced by blocking exosome formation, secretion, or uptake [43]. In our study, we found that the Alix protein level increased statistically significantly in the Aβ_1–42_ toxicity group compared to the control and vehicle groups. We found that the Alix protein level decreased statistically significantly in the Aβ_1–42_ + 25 µM ACA and Aβ_1–42_ + 50 µM ACA groups compared to the Aβ_1–42_ toxicity group. These findings show that ACA reduces exosome release. The relationship between autophagy and exosome release indicates that cellular clearance and communication processes are intricately integrated. These two processes are critical for the health and homeostasis of cells [44]. Moreover, autophagy is also one of the target pathways to combat toxic protein aggregates in AD, such as Aβ [45]. Therefore, autophagy plays an essential roles in the control of both exosomes and amyloid-β plaques. In the data we reported, we observed a significant decrease in amyloid beta levels in parallel with the decrease in Alix in the ACA treatment groups. These findings suggest a potential role for exosomes in the pathophysiology of AD and that reduced exosome release may help reduce amyloid beta dissemination. And as far as we see in our research, these findings are mediated by autophagy.

Autophagy, which occurs at the basal level in normal cells, emerges as one of the mechanisms involved in removing unnecessary and defective proteins and organelles. Induction of autophagy may occur under stress to maintain cell homeostasis [46]. According to our findings, it appears that autophagy is induced in the Aβ_1–42_ toxicity group. This suggests that Aβ_1–42_ toxicity creates stress in cells, and autophagy is induced to maintain homeostasis. However, for this process to occur smoothly, the autophagosome must combine with the lysosome to form the autolysosome structure. Considering the change in the levels of the investigated autophagic pathway proteins, it was determined that autophagy was triggered and autophagosome was formed in the Aβ_1–42_ group, but the autophagic flow was impaired due to the lack of autophagolysosome structure due to low levels of Lamp2a protein. Therefore, an increase in the amyloid-β protein and exosome-associated Alix protein level was detected with the disruption in autophagic flow interruption. In some studies, it has been reported that ACA has a neuroprotective effect, reduces amyloid-β accumulation, and slows the progression of AD [17, 40, 47, 48]. It has also been reported to trigger autophagy in different disease pathologies [19–21, 39]. Based on these literature studies, it can be said that there is a trend towards the application of ACA in the treatment of AD. We detected regulation of autophagy proteins with ACA treatment; that is, we observed that impaired autophagy returned to relatively normal conditions. In addition, it was determined that ACA reduced the amyloid-β protein level and reduced exosome release through a decrease in the Alix protein level. It is thought that immature autophagic vacuoles, which can be observed with impaired autophagy, may be the source of amyloid-β production [49, 50]. Neurons are mitotic and cannot dilute toxic substances through mitosis. Therefore, both cytoplasmic clearance and protein/organelle clearance are largely regulated by autophagy [51]. One of the main potential mechanisms in our data may be the fusion of the autophagosome with the lysosome. Degradation of Aβs in lysosomes by autophagosomal structure may reduce the level of amyloid-β protein. It has been reported in the literature that exosomes may contribute to disease progression by causing the spread of neurodegeneration-associated peptides into surrounding cells [41]. Accordingly, through the autophagy process regulated by ACA treatment, the involvement of exosomes in lysosomal degradation may reduce exosome release. A decrease in the amount of amyloid-β due to a decrease in exosome release can also be considered as a potential mechanism. In conclusion, within the scope of the current study, our research findings show that ACA treatment reduces amyloid-β and exosome release by regulating autophagy-related protein levels in Aβ peptide-induced toxicity model cells. It is thought that the decrease in Aβ and exosome levels with ACA treatment is mediated by autophagy. These findings suggest that ACA may be a potential therapeutic agent for AD.

### Limitations and Future Prospects

In conclusion, our study highlights the significant in vitro neuroprotective effect of ACA against Aβ toxicity through autophagy. Moving forward, future studies may seek to elucidate the specific neuroprotective, therapeutic effects and mechanisms of ACA via autophagy in in vivo models. Addressing the identified limitations and capitalizing on the outlined future prospects are essential steps towards harnessing the therapeutic potential of ACA in combating neurodegenerative diseases, offering renewed hope for patients and caregivers alike.

## Supplementary Information

Below is the link to the electronic supplementary material.

Supplementary file1 (DOCX 956 KB)

## Data Availability

No datasets were generated or analysed during the current study.
