# Supplementary figures and images for: Investigation of the Effects of Acacetin on Autophagy Pathway and Exosome Release in Amyloid Beta Peptide-Induced Toxicity Models
Source: Mol Neurobiol. 2025 Apr 21;62(9):11030–46. doi: 10.1007/s12035-025-04908-3 (PMC12367880; doi:10.1007/s12035-025-04908-3)

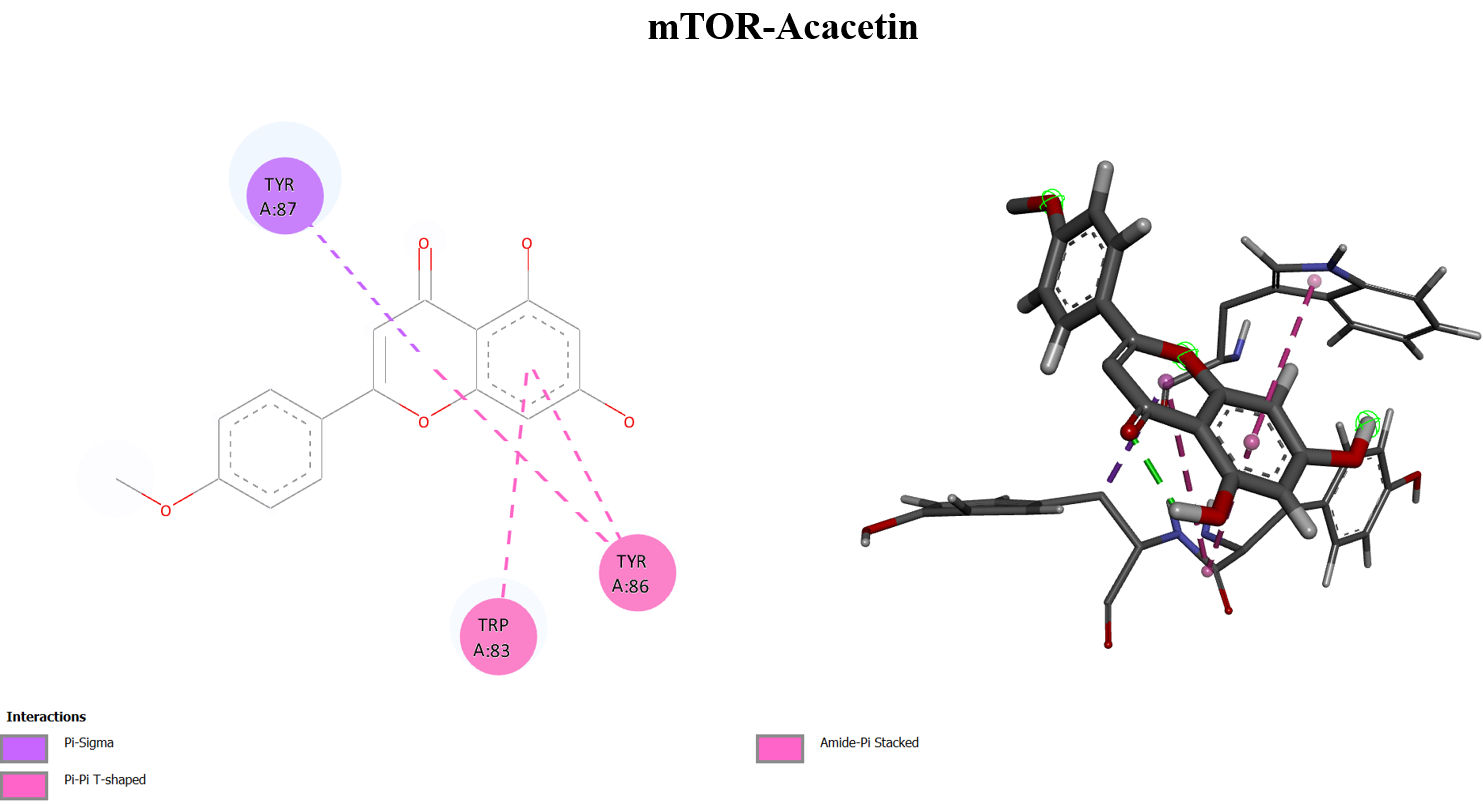


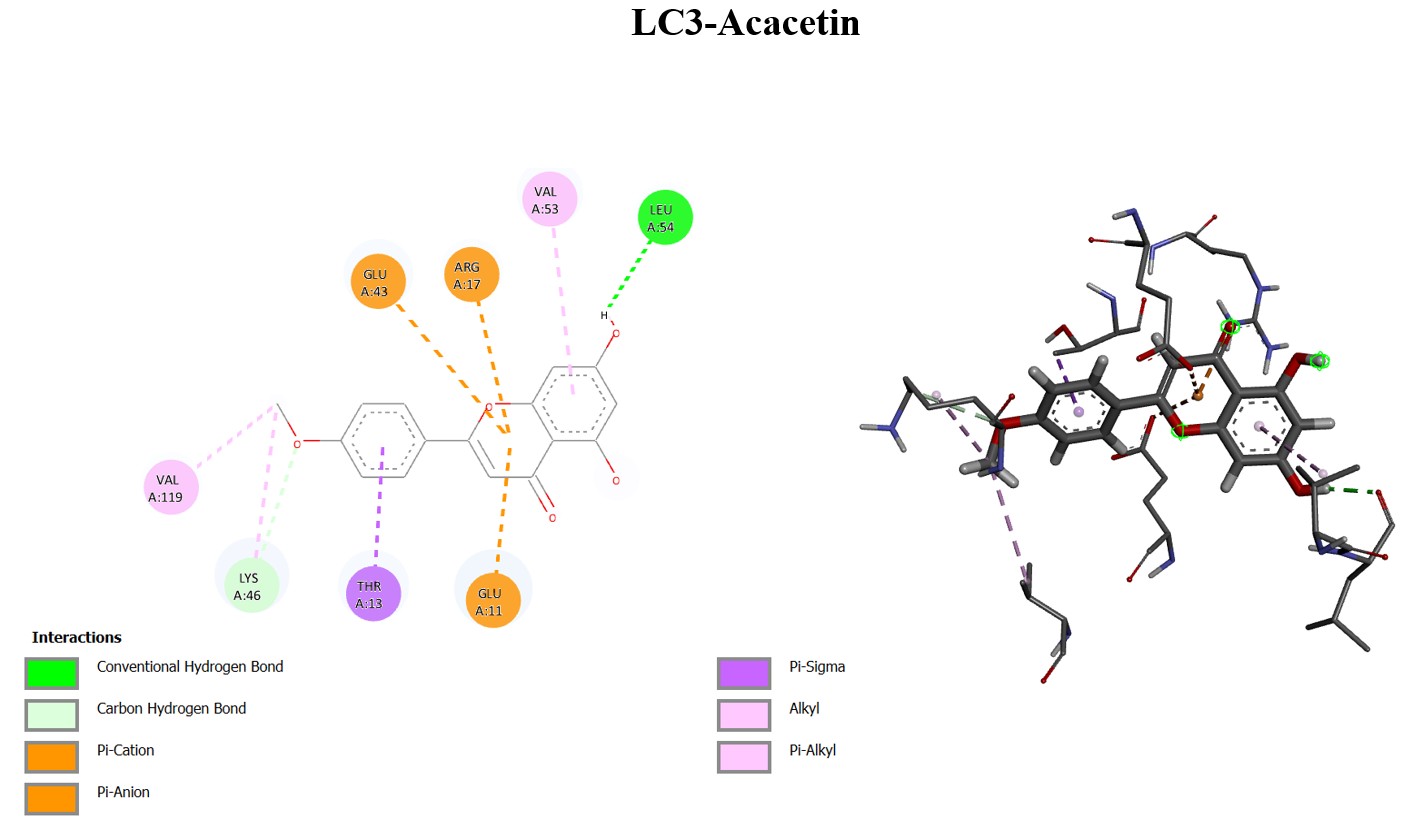


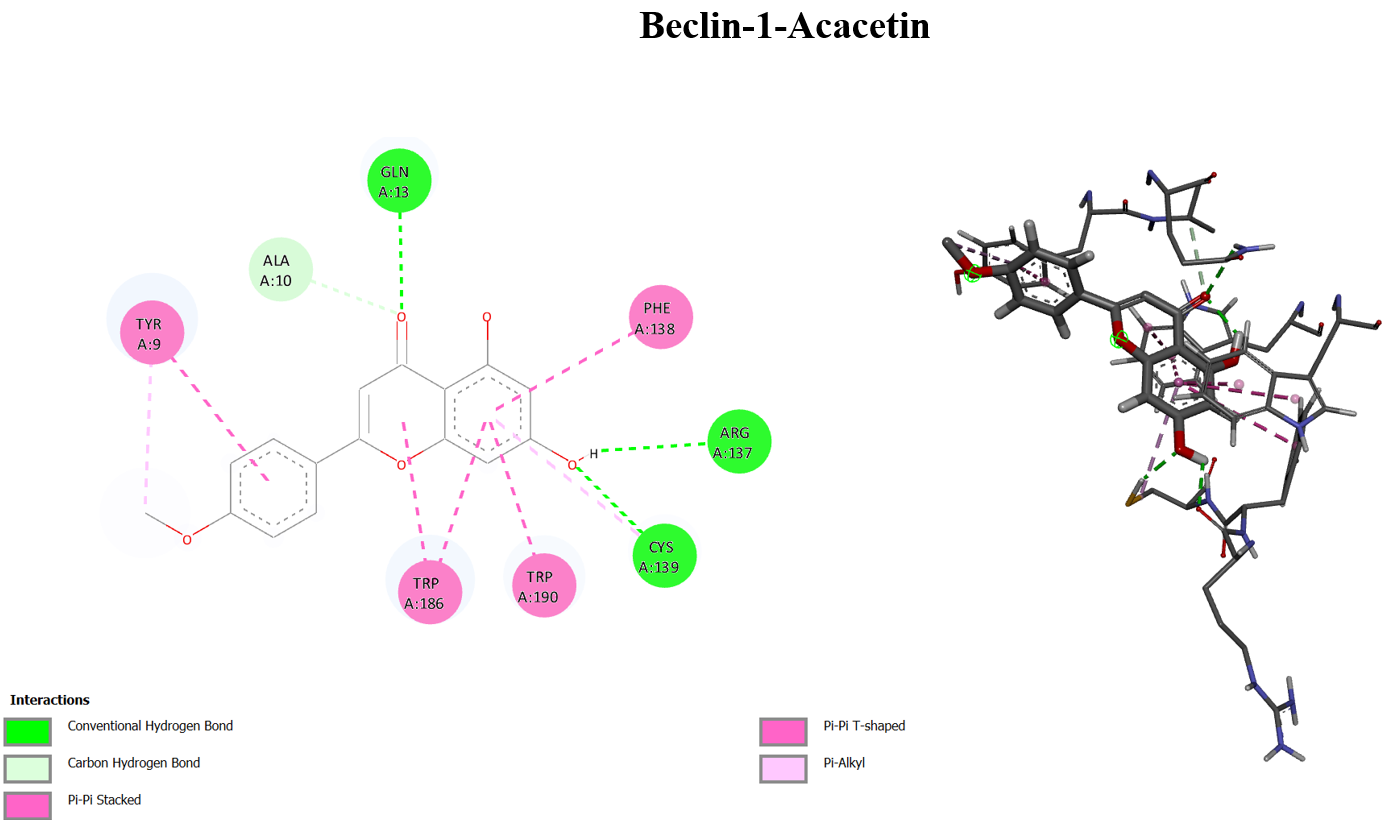


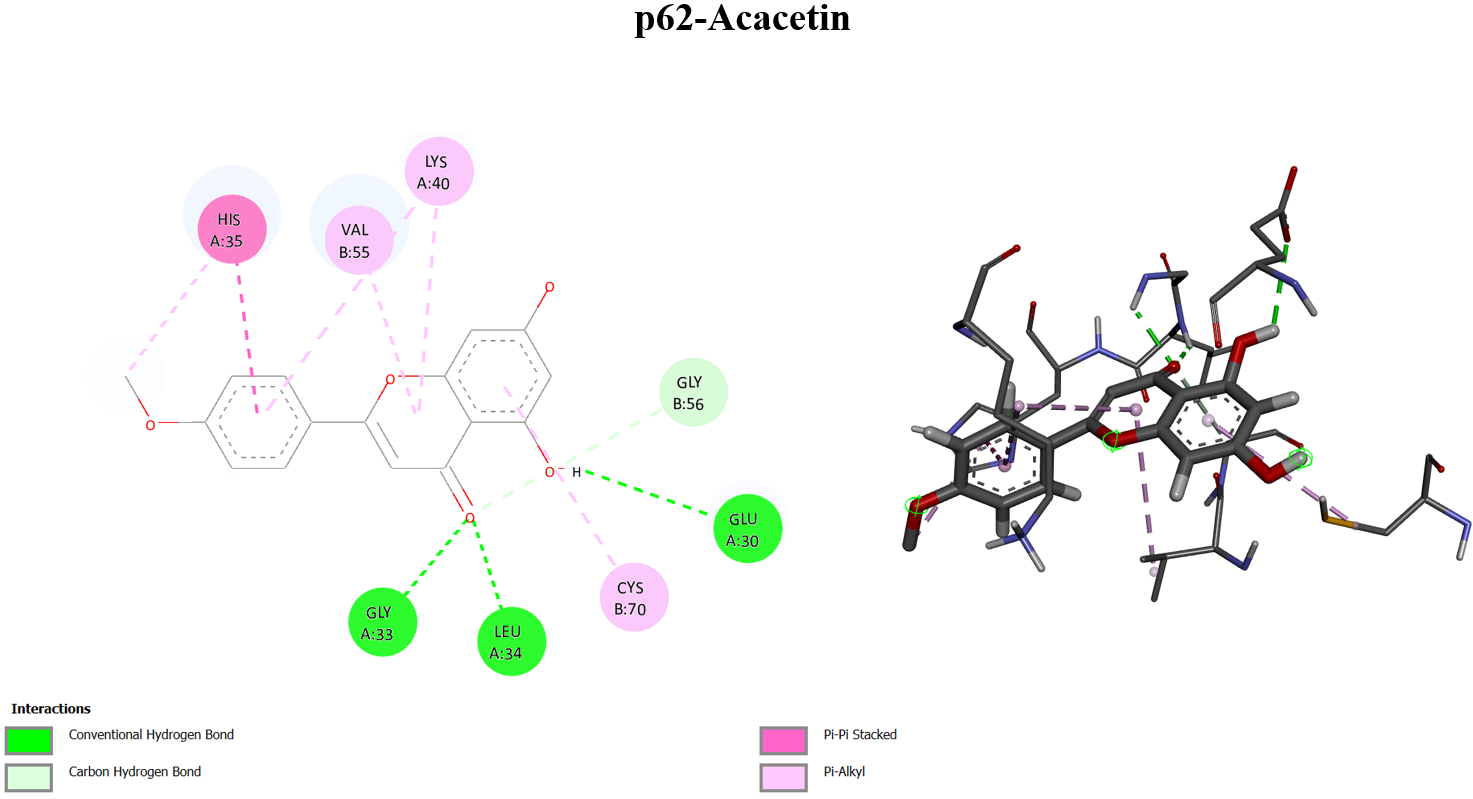


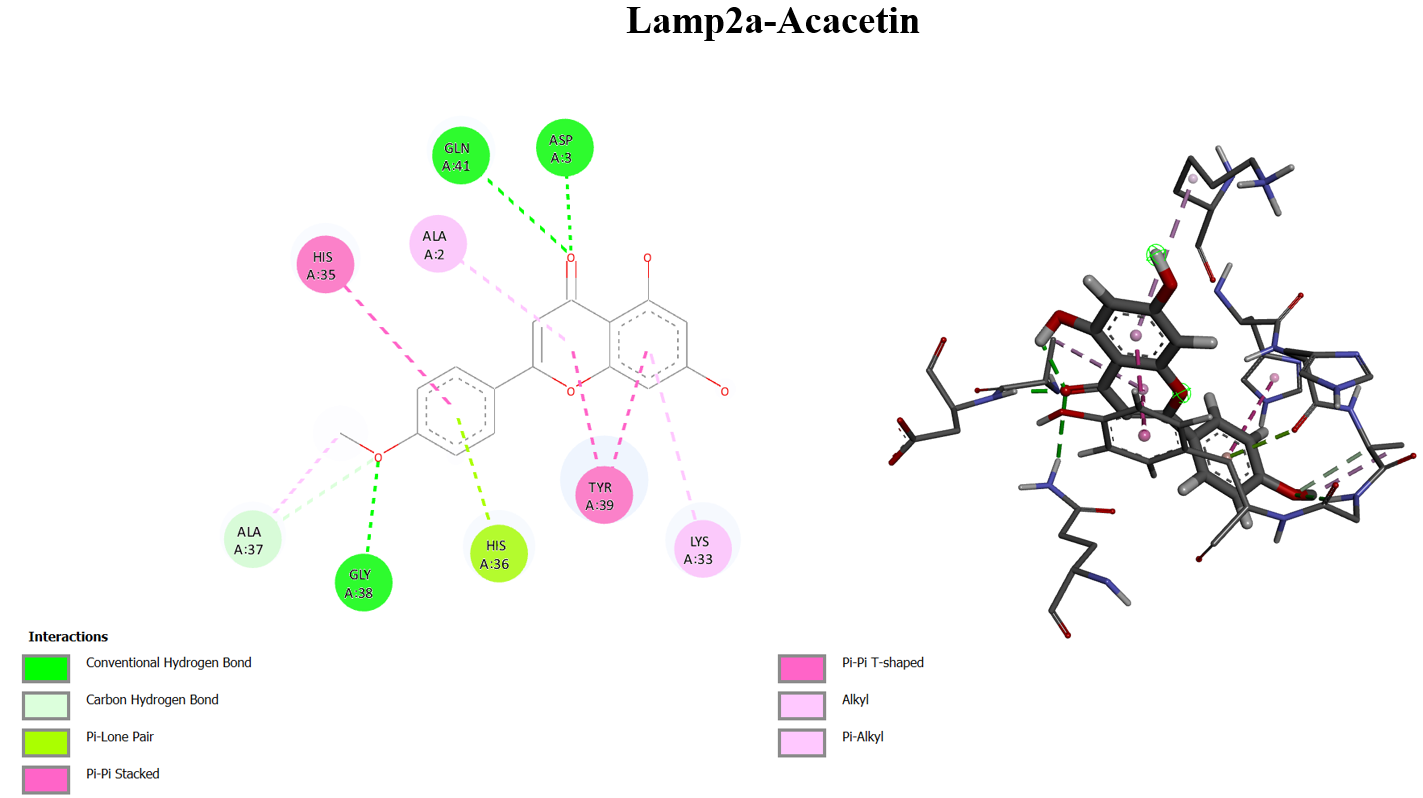

Supplement: Supplementary file 1 — Supplementary file1 (DOCX 956 KB) [file 12035_2025_4908_MOESM1_ESM.docx]
